# Prescription Department

**Published:** 1891-11

**Authors:** 


NEURALGIA AND RHEUMATISM—

| | | |
|---|---|---|
| R | Tinct. iodine comp., | |
| | Aquae ammonia | aa dr. iss. |
| | Pulv. camphor | dr. ij. |
| | Chloroform | oz. ss. |

M. Sig. Rubbed in three or four times a day.—*Dr. Baltzell.*

FOR ACUTE LARYNGITIS—

| | | |
|---|---|---|
| R | Potassi iodid | 1 dr. |
| | Potassae Chlorat Pulv. | 1 dr. |
| | Aquae destillat | 5 ozs. |
| | Syrupi | 1 oz. |

M. S. A teaspoonful every hour.—*Southern Clinic.*

IRRITABLE HEART—

The *Indiana Medical Journal* gives the following:

| | | |
|---|---|---|
| R | Tinct. pulsatillae | m xx |
| | Tinct. verat. viride | m xl |
| | Ext. Chimaphila, fl | oz. j |
| | Syr. limonis | oz. ij |

M. S. Teaspoonful before meals.

ROSSBACK'S EXPECTORANT MIXTURE—

| | |
|---|---|
| Apomorphiae hydrochlaratis | gr. i |
| Morphiae hydrochloratis | gr. ss |
| Acidi hydrochlorici | dil. gtt. x |
| Aquae destillatae | f oz. v. |

DOSE—A teaspoonful every two, three, and four hours.

The diluted hydrochlorid acid is evidently superfluous.—*American Druggist* Aug 15, 1891.

RHEUMATISM—

The following prescription is a good combination in rheumatism. I have tested it personally and know whereof I speak:

| | | |
|---|---|---|
| R | Potat. iodide | dr. iiss |
| | Tr. cimicifuga | oz. iss |
| | Vin. colch. sem. | oz. j |
| | Fl. ext. henbane | oz. ss |
| | Simple syrup | oz. v |

M. Sig. Teaspoonful well diluted with water every four hours.—*Dr. Conger in N. E. Med. Monthly.*

FLATULENCE—

Flatulence is a trouble that some times defies medical treatment. A French journal recommends the following:

R. Naphthol 1 dr.
Carb. Magnesium 1 dr.
Powd. Charcoal 1 dr.
Ess. Peppermint 2 drops

M. Sig. This is to be divided into 15 powders, and one taken at the beginning of each meal.

When the flatulence is accompanied by constipation, the following may be used:

R. Magnesium Sulphate 1 dr.
Flowers Sulphur 1 dr.

M. Sig. To be made into 15 powners, one of which is to be taken at each meal.

When diarrhoea accompanies the flatulency:

R Bicarbonate Sodium 30 grs.
Prepared Chalk 15 grs.
Powd. Nux Vomica 3 grs.

M. Sig. May be made into ten powders, one of which is given with each meal.—*Med. Brief.*

PROFUSE PURULENT EXPECTORATION—

R Ammoniac grs. 112 1-2
Aceti. sci lae grs. 225
Aquae fœniculi fl oz. 6
Ext. glycyrrhizae pur. grs 150

M. Teaspoonful every half hour. —*Med. Brief.*

ACUTE COLD—

The following is an admirable remedy for a cold of an acute character in children:

R Ammoniae mur. dr. j
Syr. scillae,
Syr. Ipecac aa dr. iij
Tr. opii. camph. dr. j
Syr. tolu q. s. ad. oz. iij

M. Sig. Take a teaspoonful every three hours.—*Atkinson.*

